# Morning glory disc anomaly with contractile movements

**DOI:** 10.1007/s00417-012-2115-4

**Published:** 2012-08-04

**Authors:** Yu Sawada, Toshiyuki Fujiwara, Takeshi Yoshitomi

**Affiliations:** Department of Ophthalmology, Akita University Graduate School of Medicine, 1-1-1 Hondo, Akita, 010-8543 Japan

## Introduction

Morning glory disc anomaly is a rare malformation characterized by an ectasia of the posterior pole of the fundus involving the optic disc. Embryologically, there are some theories for its pathogenesis, including primary mesenchymal abnormality, defective closure of the embryonic fissure, and basic defect of mesoderm combined with dynamic disturbance between the relative growth of mesoderm and ectoderm. However, the exact mechanism is still unknown. It is usually unilateral and occurs more frequently in women. Visual acuity is 20/200 or worse in 90 % of cases, but it can be as good as 20/20 [1]. Retinal detachment occurs in 30 % of cases, and many are non-rhegmatogenous with subretinal fluid accumulating around the optic disc [2]. It is usually non-contractile, but in extremely rare cases, it can exhibit contractile movements [3–5]. Here we present a contractile morning glory disc anomaly case with a video recording of the contractions.

## Case report

A 59-year-old Japanese woman had a history of poor vision in her right eye since childhood. At the age of 54, she had cataract surgery in that eye, but her vision did not improve. She was referred to our clinic for further evaluation.

On examination, her facial features were normal, exhibiting no evidence of the transsphenoidal form of basal encephalocele such as hypertelorism, a flattened nasal bridge, or a midline notch in the upper lip, commonly associated with morning glory disc anomaly. Her best-corrected visual acuity was hand motion in the right eye and 25/20 in the left eye. There was a right esotropia of 20 degrees. The pupil of the right eye was mildly dilated, and there was a relative afferent pupillary defect. The intraocular pressure was 13 mmHg in both eyes. Slit-lamp examination was unremarkable.

Funduscopic examination was normal in the left eye but disclosed a morning glory disc anomaly in the right eye. The right optic disc was excavated and enlarged. It was surrounded by an elevated and pigmented choroid. In the center of the disc there was a white membrane-like glial material overlying the cup. Retinal arterioles, some of which were sheathed and attenuated, emerged radially from the disc. The retina was largely atrophic, but there was no retinal detachment.

**Fig. 1:**
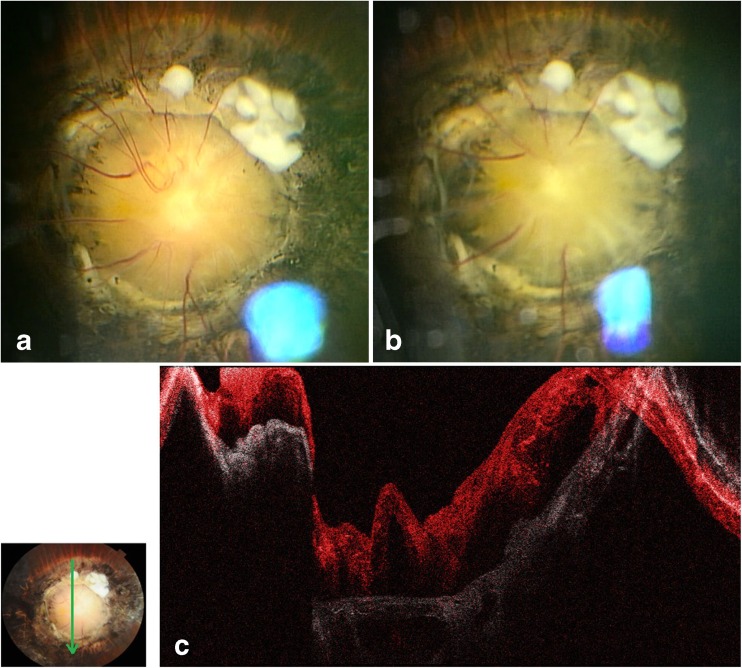
Fundus photographs of the morning glory optic disc. **a** Non-contracted phase. The optic disc was excavated and enlarged. It was surrounded by an elevated and pigmented choroid. **b** Contracted phase. When the optic disc contracted, the diameter became smaller (the *blue* color is a reflex of the contact lens.). **c** Overlapped OCT image of the contracted (*red*) and the non-contracted (*white*) phases of the morning glory optic disc. It showed that the base of the optic disc slightly moved forward during the contracted phase

With observation, the optic disc contracted and expanded in 5 to 7-s intervals with an irregular pattern (Fig. 1, supplemental video). In contraction, the diameter of the optic disc became smaller, and the retinal veins became mildly dilated and hyperemic. After the maximum contraction, the disc rapidly expanded and returned to its original shape. Contraction was provoked by strong light stimulation to the fellow eye, although not to the affected eye. It had no correlation with the respiratory cycle, Valsalva maneuver, forced eye closure, or intraocular pressure change induced by pushing on a contact lens. It was difficult to see an effect of the accommodation because the affected eye was largely esotropic. Optical coherence tomography (OCT) showed that the base of the optic disc moved slightly forward during the contraction phase (Fig. 1c).

**Fig. 2:**
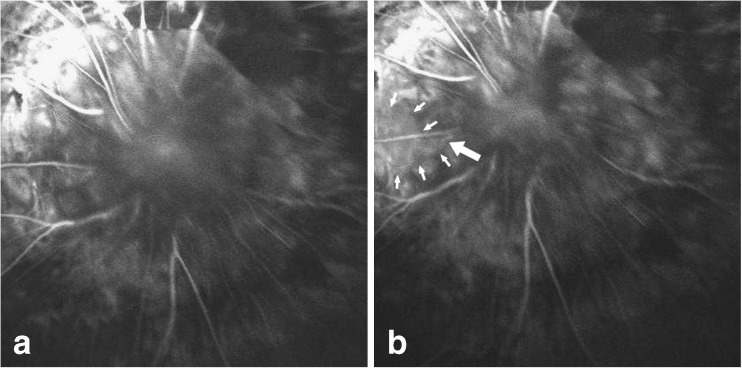
Fluorescein angiography of the right eye. **a** Before contraction. **b** When contraction occurred, some hyperfluorescent fluid was ejected from the temporal part (*thick arrow*) of the morning glory disc and floated into the vitreous cavity (*white arrows*)

Fluorescein angiography revealed a prolonged retinal circulation time of 10 s. When contraction occurred, some hyperfluorescent fluid ejected from the temporal part of the morning glory disc into the vitreous cavity (Fig. 2).

The right eye visual field was restricted to a small area of 5° in diameter in the inferior nasal area. Magnetic resonance (MR) imaging of the brain was otherwise normal except for esotropia and posterior elongation of the right eye. It did not show brain malformations such as the transsphenoidal form of basal encephalocele or agenesis of the corpus callosum. MR angiography revealed no sign of intracranial vascular anomalies including moyamoya disease. The patient experienced no change over 6 months of follow-up.

## Discussion

Congenital optic disc excavated disorders include optic disc coloboma, peripapillary staphyloma, and morning glory disc anomaly. Rarely are they associated with the optic disc contractility. While there are distinct differences among these disorders both clinically and embryologically, the proposed pathogenesis of the contraction movements shares some similarity.

The possible mechanisms of the movements are classified into two groups: pressure balance and muscular contraction [6]. In a case reported by Sugar and Beckman, the contraction was related to the respiratory cycle and followed changes in venous pressure [7]. An alternative pressure balance mechanism hypothesis proposes that there is an anomalous communication between the subarachnoid space and the juxtapapillary subretinal space [8]. Accordingly, changes in transient pressure gradients occur between the two compartments, causing the flow of fluid back and forth along the optic nerve, thus causing the contraction and expansion.

The muscle contraction mechanism has been proposed by several authors. Wise et al. suggested that the contractile movement is due to the presence of an atavistic retractor bulbi muscle lying alongside the optic nerve [6]. This muscle could pull directly on the staphyloma, and squeeze it within a cone of muscle. The contraction could be evoked by forced eyelid closure and light-induced squinting of the normal eye. Lee et al. proposed another muscle-related mechanism in which heterotopic smooth muscle in the posterior sclera would contract under the influence of parasympathetic cholinergic neurons [4]. The heterotopic muscle was considered to be a ciliary muscle because the contraction was provoked by strong light stimulation and accommodation effort. Kral and Svarc suggested a sphincter iridis muscle, as the contraction was evoked by light stimulation and ended in a hippus-like movement, simulating pupillary movement [9].

Our case favors the ectopic cholinergic muscle contraction mechanism because the movement was provoked by strong light stimulation to the fellow eye, which is similar to the reaction of the ciliary muscle and sphincter iridis muscle. Light stimulation to the affected eye failed to provoke contraction, probably because the optic nerve was already severely damaged. The pressure balance mechanism is not a likely explanation in this case because there was no peripapillary subretinal fluid, and the contraction was not related to the respiratory cycle.

Another interesting feature of this case is that the fluorescein angiography showed some fluid being ejected from the optic disc into the vitreous cavity when the contraction occurred. There are previous reports indicating a communication between the perineural space and the vitreous cavity in morning glory disc anomaly and optic disc pit [2, 10]. In our case, the fluid of the perineural space looked to be pushed out as the contraction shrunk the perineural space and increased the pressure. This feature might be proof of the communication between these two spaces.

In conclusion, we report a case of morning glory disc anomaly with contractile movement. The extraordinary features described here can help ophthalmologists to recognize this rare anomaly and better understand it.

## Electronic supplementary materials

Below is the link to the electronic supplementary material.

ESM 1 (MPG 103348 kb)

## References

[1] Beyer WB, Quencer RM, Osher RH (1982). Morning glory syndrome. a functional analysis including fluorescein angiography, ultrasonography, and computerized tomography. Ophthalmology.

[2] Haik BG, Greenstein SH, Smith ME, Abramson DH, Ellsworth RM (1984). Retinal detachment in the morning glory anomaly. Ophthalmology.

[3] Cennamo G, Crecchio G, Iaccarino G, Forte R, Cennamo G (2010). Evaluation of morning glory syndrome with spectral optical coherence tomography and echography. Ophthalmology.

[4] Lee JE, Kim KH, Park HJ, Lee SJ (2009). Morning glory disc anomaly: a computerized analysis of contractile movements with implications for pathogenesis. J AAPOS.

[5] Brodsky MC (2006). Contractile morning glory disc causing transient monocular blindness in a child. Arch Ophthalmol.

[6] Wise JB, Maclean AL, Gass DM (1966). Contractile peripapillary staphyloma. Arch Ophthalmol.

[7] Sugar HS, Beckman H (1969). Peripapillary staphyloma with respiratory pulsation. Am J Ophthalmol.

[8] Golnik KC (2008). Cavitary anomalies of the optic disc: neurologic significance. Curr Neurol Neurosci Rep.

[9] Kral K, Svarc D (1971). Contractile peripapillary staphyloma. Am J Ophthalmol.

[10] Irvine AR, Crawford JB, Sullivan JH (1986). The pathogenesis of retinal detachment with morning glory disc and optic pit. Retina.

